# Phenotypes, antioxidant responses, and gene expression changes accompanying a sugar-only diet in *Bactrocera dorsalis* (Hendel) (Diptera: Tephritidae)

**DOI:** 10.1186/s12862-017-1045-5

**Published:** 2017-08-17

**Authors:** Er-Hu Chen, Qiu-Li Hou, Dan-Dan Wei, Hong-Bo Jiang, Jin-Jun Wang

**Affiliations:** Key Laboratory of Entomology and Pest Control Engineering, College of Plant Protection, Southwest University, Chongqing, 400715 People’s Republic of China

**Keywords:** *Bactrocera dorsalis*, Stress resistance, Ovary development, Gene expression, Oxidative damage, Sugar-only diet

## Abstract

**Background:**

Diet composition (yeast:carbohydrate ratio) is an important determinant of growth, development, and reproduction. Recent studies have shown that decreased yeast intake elicits numerous transcriptomic changes and enhances somatic maintenance and lifespan, which in turn reduces reproduction in various insects. However, our understanding of the responses leading to a decrease in yeast ratio to 0% is limited.

**Results:**

In the present study, we investigated the effects of a sugar-only diet (SD) on the gene expression patterns of the oriental fruit fly, *Bactrocera dorsalis* (Hendel), one of the most economically important pests in the family Tephritidae. RNA sequencing analyses showed that flies reared on an SD induced significant changes in the expression levels of genes associated with specific metabolic as well as cell growth and death pathways. Moreover, the observed upregulated genes in energy production and downregulated genes associated with reproduction suggested that SD affects somatic maintenance and reproduction in *B. dorsalis*. As expected, we observed that SD altered *B. dorsalis* phenotypes by significantly increasing stress (starvation and desiccation) resistance, decreasing reproduction, but did not extend lifespan compared to those that received a normal diet (ND) regime. In addition, administration of an SD resulted in a reduction in antioxidant enzyme activities and an increase in MDA concentrations, thereby suggesting that antioxidants cannot keep up with the increase in oxidative damage induced by SD regime.

**Conclusions:**

The application of an SD diet induces changes in phenotypes, antioxidant responses, and gene expressions in *B. dorsalis*. Previous studies have associated extended lifespan with reduced fecundity. The current study did not observe a prolongation of lifespan in *B. dorsalis*, which instead incurred oxidative damage. The findings of the present study improve our understanding of the molecular, biochemical, and phenotypic response of *B. dorsalis* to an SD diet.

**Electronic supplementary material:**

The online version of this article (doi:10.1186/s12862-017-1045-5) contains supplementary material, which is available to authorized users.

## Background

Nutrients are critical environmental signals that influence growth and development in insects [1]. Macronutrient balance is an important determinant of fitness, and previous studies have shown that altering the concentrations of yeast and sugar has a profound impact on the lifespan of *Drosophila melanogaster*, thereby suggesting that dietary protein:carbohydrate (P:C) balance is the main driver of lifespan and ageing processes [2–4]. In addition, yeast is a complex mixture of nutrients, and lifespan differences between diets with small amounts of yeast or no yeast are explained by deficiencies in both protein and micronutrients in *Bactrocera tryoni* [5]. In many tephritid flies, a diet combining sugar with yeast hydrolysate, which is a rich source of carbohydrates and proteins, has been shown to enhance reproductive development, sexual performance, fecundity, and lifespan in comparison with a diet of sugar alone [6]. Moderate restriction of protein in the adult diet extends the lifespan of various species ranging from yeast to primates, and the explanations for lifespan extension by dietary protein restriction assumed an adaptive allocation of limited resources between reproductive effort and somatic repair [7]. A higher ratio of protein to carbohydrate in adult diets has been associated with shorter lifespan in insects, thereby suggesting that protein overconsumption is toxic [8]. Dietary proteins are essential for the growth and development of organisms, and a previous study has shown that *D. melanogaster* fed on a sugar-only diet (SD) can lead to the inhibition of cell growth and cell cycle progression [9].

Life-history traits are often negatively associated with each other and thus play an important role in insect development [10]. Research investigations have described a trade-off between fecundity and longevity and qualitative and quantitative changes in the diets of multicellular organisms, including *Anastrepha ludens*, *Trichopria drosophilae*, and *D. melanogaster* [11–13]. Different diet sources vary in nutritional content, thereby influencing diverse biochemical pathways. Studies have been conducted to underlie the genetic and molecular mechanisms involved in different dietary status, which will assist in bettering understand the relationship among phenotype, gene expression, and the environment [14, 15].

Reactive oxygen species (ROS) are by-products of aerobic metabolism that occurs in the mitochondria that induce oxidative damage to cell, which in turn causes aging and eventually death [16]. Therefore, the activities of different antioxidant enzymes may have a significant effect on the lifespan of organisms. For example, the overexpression of superoxide dismutase (SOD), a major cytosolic enzyme responsible for scavenging highly toxic superoxide radicals in certain tissues, increases the lifespan of *Drosophila* [17]. On the contrary, knocking out the SOD gene dramatically shortens lifespan of *D. melanogaster* to a few days by inducing higher levels of oxidative damage [18]. Protein restriction has been demonstrated to reduce the amount of oxidative damage of DNA and other major cellular components. For example, the activities of SOD and catalase (CAT) in liver homogenates of 12- and 24-month-old mice increase with protein restriction [19]. The mechanisms underlying lifespan extension by protein restriction may be the upregulation of SOD activity, which is associated with the insulin signaling pathway [20]. Insulin belongs to a superfamily of peptide hormones, which include relaxins, insulin-like peptides (ILPs) and insulin-like growth factors (IGFs). The insulin signaling pathway plays a central role in nutrition regulated growth [21]. In response to dietary proteins, ILPs function in an endocrine manner and trigger growth by binding to insulin receptors (InRs) and subsequently activating a conserved PI3 kinase (PI3K) and Akt kinase signaling pathways in all tissues of *D. melanogaster* [22].

The oriental fruit fly, *Bactrocera dorsalis* (Hendel) (Diptera: Tephritidae), is one of the most important pests of fruits and vegetables in South East Asia and the Pacific region [23]. It is highly polyphagous, infesting a wide variety of fruit crops such as citrus, mandarin, peach, and mango [24], and induces significant economic losses through direct fruit damage, fruit drop, and export limitations associated with quarantine restrictions. Moreover, diet is one of the most important environmental variables that affects its growth, reproduction, and distribution; therefore, the ability to adapt to different dietary conditions is critical for their survival [25]. Several studies have investigated the phenotypic response of flies to different nutritional diets; however, the effects of SD on the phenotype and gene expression in *B. dorsalis* remain unclear. In the present study, gene expression, ovary development, lifespan, somatic maintenance (starvation and desiccation resistance), antioxidant enzyme activities (SOD, glutathione S-transferase (GST), peroxidase (POD), and CAT), the total antioxidant capacity (T-AOC), and malondialdehyde (MDA) content were examined in *B. dorsalis* after exposure to an SD or a normal diet (ND) regimen. The aim of the present study was to facilitate a better understanding the phenotypes, antioxidant responses, and gene expression patterns in response to the SD regimen in *B. dorsalis*.

## Methods

### Insect

*B. dorsalis* was collected and reared as previously described [26]. Briefly, the flies were reared in plastic cages at 27 ± 1 °C, 70 ± 5% relative humidity, and a photoperiod of 14:10 (L:D) h.

### Experimental diets

Variable amounts of yeast extract (Oxoid Ltd., Basingstoke, Hampshire, England) per 100 g of adult food (yeast:sucrose) were used in this study, as well as a sugar-only diet (yeast:sucrose = 0:100, 0% yeast), and normal diet (yeast:sucrose = 25:75, 25% yeast). Yeast mainly comprised 62.5% protein, as well as water soluble B-complex vitamins, and sodium chloride, and had a pH of 7.0 ± 0.2 (0.5% solution), and temperature of 25 °C. The *B. dorsalis* were given ad libitum access to all diets, assuming that sucrose in each of the diets is not limiting, and that any response to SD is primarily mediated through variations in yeast content.

### Insect samples and RNA extraction

*B. dorsalis* were reared at a density of 15 males and 15 females per jar and randomly assigned to either the SD or ND diet. Thirty-day-old female flies from each diet group were collected. Three replicates were performed for each diet. RNA was extracted using the RNeasy plus Micro Kit (Qiagen GmbH, Hilden, Germany), following the manufacturer’s instructions. RNA was quantified by measuring the absorbance at a wavelength of 260 nm using a NanoVue UV-Vis spectrophotometer (GE Healthcare Bio-Science, Uppsala, Sweden). The purity of all RNA samples was assessed at an absorbance ratio of OD_260/280_ and OD_260/230_, and the integrity of RNA was confirmed by 1% agarose gel electrophoresis.

### cDNA library construction and sequencing

Briefly, oligo (dT) magnetic beads were used to select mRNAs with a poly(A) tail, and the target RNA was obtained after purification. First-strand cDNA was synthesized using random hexamer-primers from purified poly(A) mRNA. Second-strand cDNA was synthesized using buffer, dNTPs, RNaseH, and DNA polymerase I. Short fragments were purified using a QiaQuick PCR extraction kit. These fragments were washed with ethidium bromide buffer for end reparation poly(A) addition and then ligated to sequencing adapters. Suitable fragments, as judged by agarose gel electrophoresis, were selected as templates for PCR amplification. The resulting cDNA library was sequenced on a next-generation sequencing platforms (complete genomics) using paired-end technology in a single run.

### Analysis of RNA-seq data

Raw reads produced in this study were cleaned by discarding reads with adapters and reads in which unknown bases comprised more than 10%. Low quality reads (a percentage of low quality bases over 50% in a read) were also excluded from further analysis. HISAT was used to map clean reads to the *B. dorsalis* genome reference and Bowtie2 [27] to the gene reference using the default parameters, respectively. Gene expression levels in terms of transcripts were quantified by RSEM (RNA-Seq by Expectation Maximization) and FPKM (Fragment Per Kilobase of exon model per million mapped reads) method [28]. The FPKM between the biological replications was analyzed by Pearson correlation. The Pearson coefficient of gene expression in different replications was more than 0.85, indicating consistency between the replicates. When the value of either sample FPKM was zero, 0.01 was used to instead of 0 to calculate the fold change. According to the correlation results, the NOISeq method was selected to analyze differential expression between the SD and ND diets [29]. We used the absolute value of log_2_ ratio ≥ 1 and probability ≥0.8 as the threshold to judge significant differences in gene expression [30].

Gene Ontology (GO) functional analysis provides GO functional classification annotation of DEGs as well as GO functional enrichment analysis. The annotation terms formed the GO ontology and were obtained from Blast2GO [31]. This method first maps all DEGs to GO terms in the database (www.geneontology.org), calculating gene numbers for every term, then uses a hypergeometric test to find significantly enriched GO terms in the input list of DEGs based on ‘GO::TermFinder’ (http://www.yeastgenome.org/help/analyze/go-term-finder). The *p*-value for the hypothesis test was calculated using the following formula:

$$ p=1-\sum_{i=0}^{m-1}\frac{\left(\begin{array}{c}\hfill M\hfill \\ {}\hfill i\hfill \end{array}\right)\left(\begin{array}{c}\hfill N-M\hfill \\ {}\hfill n-i\hfill \end{array}\right)}{\left(\begin{array}{c}\hfill N\hfill \\ {}\hfill n\hfill \end{array}\right)}, $$

where *N* is the number of all genes with a GO annotation; *n* is the number of DEGs in *N*; *M* is the number of all genes that were annotated to certain GO terms; *m* is the number of DEGs in *M*. The calculated *p*-value was subjected to Bonferroni correction, taking the corrected *p*-value <0.05 as a threshold. GO terms fulfilling this condition were defined as significantly enriched GO terms in DEGs.

Pathway analysis helps in further elucidating the distinct functions of gene regulation and identify the significant pathways of the DEGs according to the Kyoto Encyclopedia of Genes and Genomes (KEGG) database, which is a major public database on pathway enrichment analysis [32]. The formula for the *p*-value is similar to that of the GO analysis. Here, *N* is the number of all genes with KEGG annotation, *n* is the number of DEGs in *N*, *M* is the number of all genes annotated to specific pathways, and *m* is the number of DEGs in *M*.

### Effect of SD diet on ovary development

For the SD and ND diets, five jars (100 mm high × 70 mm diameter) were each set up for 10 females and 10 males, making a total of 10 jars (*n* = 50 females per diet). Food and water were both supplied from two small vessels and placed in each jar. Food was replaced every 7 days, and water was replaced daily.

To maintain a 1:1 sex ratio in each jar, dead males were replaced with age-matched males until the female adult died. For ovary development, 30-day-old female flies from each jar were dissected, and the maximum diameter of each entire ovary was measured. The images were captured with a Leica M205A stereomicroscope (Leica Microsystems, Wetzlar, Germany).

### Starvation and desiccation treatment

For the SD and ND diets, five jars (100 mm high × 70 mm diameter) were each set up for 10 females and 10 males, making a total of 10 jars (*n* = 50 females per diet). Diet and water were both supplied from two small vessels and placed in each jar. To maintain a 1:1 sex ratio in each jar, dead males were replaced with age-matched males. When a female died, a male was also removed. For starvation diet, the female flies that emerged in each jar at day were transferred into a new jar containing a small vessel plugged with cotton to prevent desiccation. After transferring the flies, the number of dead flies was recorded each day until all were dead. For desiccation diet, the female flies that emerged in each jar at day 30 were transferred into a new jar containing a disc of dry filter paper. Desiccation jars were observed every 12 h after transferring the flies until all were dead.

### Effect of SD diet on fly lifespan

For the SD and ND diets, we prepared 10 jars (100 mm high × 70 mm diameter) of 5 females and 5 males, making a total of 20 jars (*n* = 50 females per diet). Flies were maintained at a 1:1 sex ratio. When a female died, an opposite-sex individual was removed, and when a male died, it was replaced with an age-matched individual of the same sex. The flies were supplied with food and water ad libitum using the food and water-vessel system as previously described.

### Protein extraction

Protein extraction was conducted using a commercially available assay kit (Nanjing Jiancheng Bioengineering Institute, Jiangsu, China). The treated females were first homogenized in 0.9% saline at a ratio of 1:9 (W_flies_:V_saline_). The crude homogenates were centrifuged at 4 °C and 10,000 *g* for 10 min. The supernatants were collected and stored on ice until assayed. The protein concentration was determined using the Bradford (1976) [33] method, with bovine serum albumin as standard.

### Enzyme activity, T-AOC, and lipid peroxidation (LPO) assay

Assay kits for SOD (Item number: A001–3), POD (Item number: A084–1), CAT (Item number: A007–2), T-AOC (Item number: A015–1), and MDA (Item number: A003–1) were used in this study (Nanjing Jiancheng Bioengineering Institute, Nanjing, China). Absorbances were read in a microplate spectrophotometer (XMark™, Bio-Rad, Hercules, CA, USA). Specifically, SOD activity was determined at a wavelength of 550 nm using xanthine and xanthine oxidase systems. One unit of SOD activity was defined as the amount of enzyme required to cause 50% inhibition of xanthine oxidase in 1 mL of enzyme extraction of 1 mg protein. POD activity was determined at a wavelength of 420 nm by catalyzing the oxidation in the presence of H_2_O_2_ as a substrate. One unit of POD activity was defined as the amount that catalyzes 1 μg of substrate per min per mg protein. CAT activity was calculated by measuring the decrease in absorbance at a wavelength of 240 nm due to H_2_O_2_ decomposition. One unit of CAT activity was defined as the amount that decomposes 1 μmol of H_2_O_2_ per sec per mg protein. GST activity was determined with 1-chloro-2,4-dinitrobenzene (CDNB, Shanghai Chemicals, Shanghai, China) and reduced GSH (Sigma-Aldrich, St. Louis, MO, USA) as substrates according to Habig et al. (1974) [34], with slight modifications. Briefly, a 96-well microplate with 100 μL CDNB (1% ethanol (*V*/V) included) and 100 μL GSH in 0.9% saline in each well was incubated for 20 min at 37 °C. Subsequently, 100 μL of each enzyme solution was added to individual wells to a final concentration of 0.2 mM CDNB and 2.0 mM GSH. Absorbance was measured continuously at a wavelength of 340 nm and 37 °C for 5 min. Absorbance changes per min were converted into nmol CDNB conjugated/min/mg protein using the extinction coefficient of the resulting 2,4-dinitrophenyl-glutathione: ε_340 nm_ = 9.6 mM^−1^ cm^−1^ [34].

T-AOC was measured based on the generation of the Fe^2+^-o-phenanthroline complex, as the overall reducing agents in the sample supernatant that reduced Fe^3+^ to Fe^2+^ in the sample supernatant. The absorbance of the Fe^2+^-o-phenanthroline complex was measured at a wavelength of 520 nm. One unit of T-AOC was defined as the amount necessary to increase absorbance by 0.01 per min per mg protein. LPO levels were indirectly determined by using MDA levels. MDA production was evaluated by reacting with thiobarbituric acid to yield a red species with a maximum absorbance at 532 nm. The MDA concentration was expressed as nmol of MDA produced per mg protein.

### Statistical analysis

Data were analyzed using SPSS 16.0 software (SPSS Inc., Chicago, IL, USA, 2008) and are presented as the mean ± SE. Significant differences between treatments were analyzed by using an independent Student’s *t*-test (for comparison of two means) (* *P* < 0.05, ** *P* < 0.01, *** *P* < 0.001).

## Results

### Evaluation of RNA-seq data

In the present study, samples from *B. dorsalis* were sequenced using the RNA-seq technology. More than 99.98% of the clean data was obtained from each sample for pair-end sequencing, and the length of sequence reads was 50 bp (Additional file 1: Table S1). To evaluate the quality of the sequencing data, several aspects of each *B. dorsalis* sample were subjected to strict quality control and the average genome mapping ratio was 83.22% (Additional file 2: Table S2). The FPKM density had a similar pattern in each sample of *B. dorsalis*, indicating that the transcriptome analysis of each diet was highly reproducible (Additional file 3: Figure S1).

### Analysis of gene expression profiling

The gene expression profiles of the flies fed on an SD or ND were analyzed at day 30 after emergence of *B. dorsalis*. A total of 2600 (1656 downregulated, 944 upregulated) DEGs were generated between the SD and ND groups, and the magnitude of these changes were defined as abs (log2(Y/X) ≥ 1 and divergence probability ≥0.8) (Additional file 4: Figure S2 and Table S3).

For GO analysis, DEGs were divided into three ontologies: biological process, cellular component, and molecular function, including 51 annotations (Additional file 5: Figure S3A). The majority of the enriched and upregulated genes were assigned into the following GO categories (Additional file 6: Table S4): ‘generation of precursor metabolites and energy’ (10.7%, *P* = 1.15e-23), ‘energy derivation by oxidation of organic compounds’ (9.8%, *P* = 5.54e-23), ‘ATP metabolic process’ (2.9%, *P* = 9.23e-08), ‘glycosyl compound biosynthetic process’ (2.9%, *P* = 2.68e-07), ‘somatic muscle development’ (1.6%, *P* = 0.0088), and ‘aerobic respiration’ (2.5%, *P* = 0.035). Most of the downregulated genes were included in the ‘chromosome organization’ (10.1%, *P* = 2.71e-20), ‘cell cycle’ (17%, *P* = 6.48e-20), ‘organelle organization’ (18.4%, *P* = 8.01e-07), ‘negative regulation of metabolic process’ (6.3%, *P* = 9.10e-07), ‘reproduction’ (16%, *P* = 4.99e-06), and ‘female gamete generation’ (9.1%, *P* = 6.93e-05) categories (Additional file 7: Table S5).

**Fig. 1:**
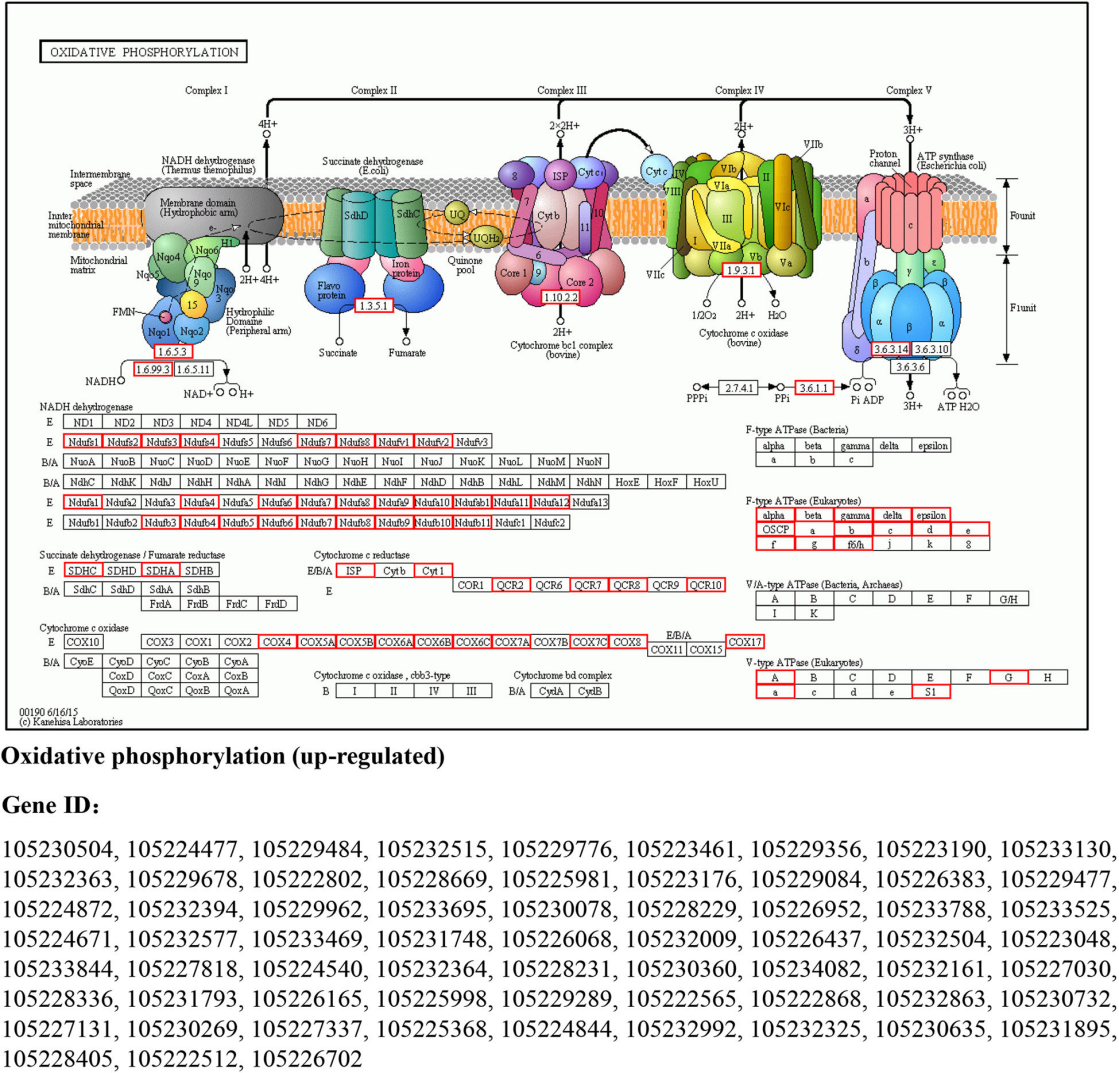
The KEGG pathway of oxidative phosphorylation responds to the chronic sugar-only diet regime. Genes highlighted in red are enriched and upregulated under sugar-only diet. The Bonferroni correction method was used for multiple hypothesis test correction and FDR-corrected *P* < 0.05 as cut-off

**Fig. 2:**
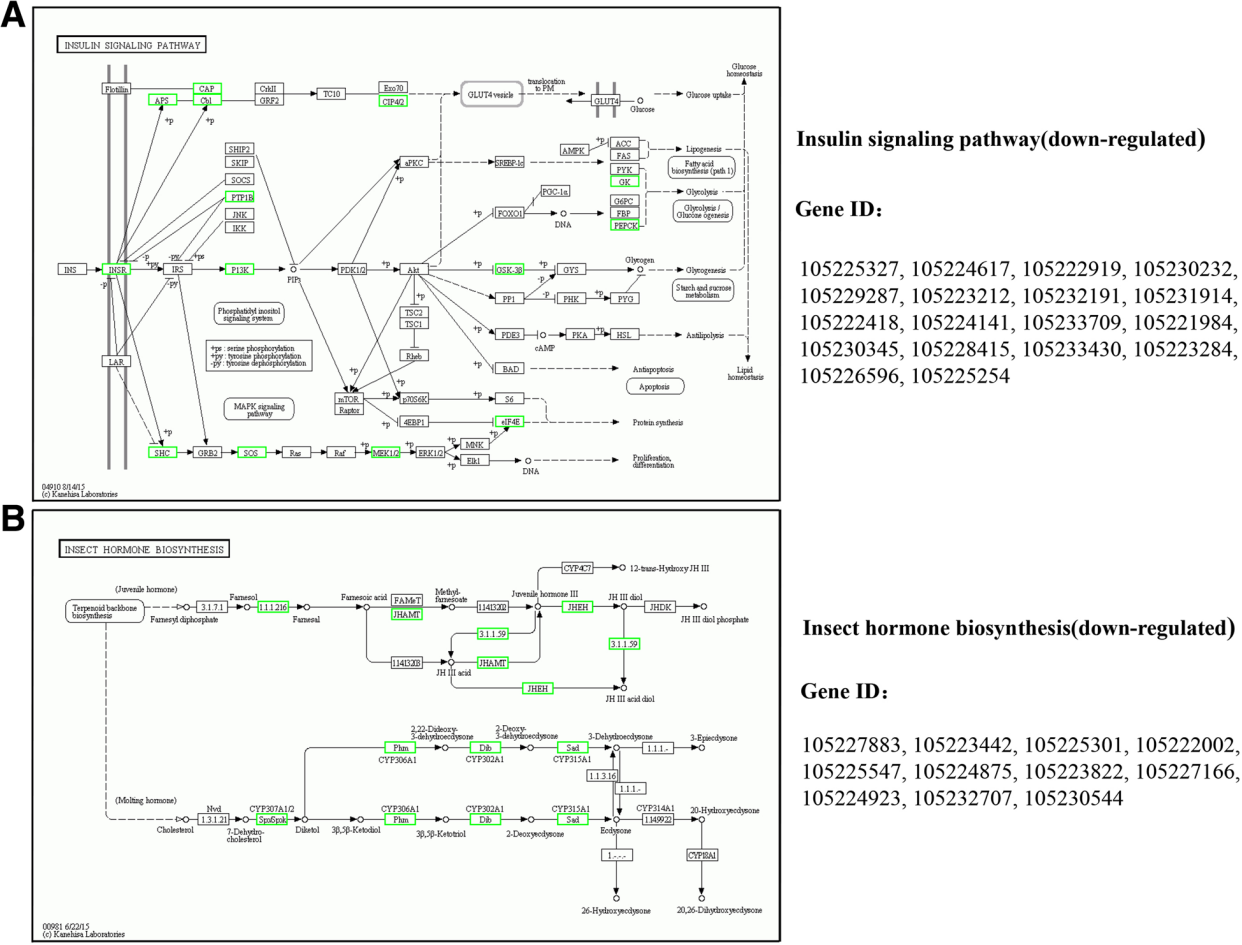
**a** Downregulated genes involved in the insulin signaling pathway in response to the sugar-only diet. **b** Down-regulated genes involved in hormone synthesis signaling pathway response to the sugar-only diet. Genes highlighted in green are enriched and down-regulated under sugar-only diet. The Bonferroni correction method was used for multiple hypothesis test correction and FDR-corrected *P* < 0.05 as cut-off

For KEGG analysis, the DEGs were enriched into 32 KEGG pathways, including the categories “cellular process”, “environmental information processing”, “genetic information processing”, “metabolism”, and “organismal system” (Additional file 5: Figure S3B). The upregulated genes were assigned to the following KEGG pathways: namely, ‘oxidative phosphorylation’ (9.9%, *P* = 3.81e-41) (Fig. 1), ‘metabolic pathways’ (23.8%, *P* = 2.41e-10), ‘calcium signaling pathway’ (3.7%, *P* = 2.86e-06), ‘MAPK signaling pathway’ (4.3%, *P* = 2.82e-05), ‘folate biosynthesis’ (1.35%, *P* = 0.000292), ‘biosynthesis of amino acids’ (2.1%, *P* = 0.00158), ‘alanine, aspartate and glutamate metabolism’ (1.2%, *P* = 0.00281), ‘drug metabolism cytochrome P450’ (2.0%, *P* = 0.0056), ‘cAMP signaling pathway’ (3.1%, *P* = 0.0068), and ‘cysteine and methionine metabolism’ (1.2%, *P* = 0.0327) (Table 1). The downregulated genes were enriched in the ‘cell cycle’ (4.0%, *P* = 9.25e-19), ‘insulin signaling pathway’ (1.4%, *P* = 0.015) (Fig. 2a), ‘the target of rapamycin (TOR) signaling pathway’ (0.41%, *P* = 0.0042) (Additional file 8: Figure S4), ‘insect hormone biosynthesis (including juvenile hormone (JH) biosynthesis)’ (0.90%, *P* = 0.036) (Fig. 2b), and ‘p53 signaling pathway’ (0.98%, *P-*values = 0.012) categories (Table 2).

**Table 1:** Selected KEGG pathways significantly enriched for up-regulated genes in sugar-only diet (SD) versus normal diet (ND)

KEGG pathway	Pathway ID	DEGs (668)	*P*-value	Level 1	Level 2
Oxidative phosphorylation	ko00190	66 (9.88%)	3.81e-41	Metabolism	Energy metabolism
Metabolic pathways	ko01100	159 (23.8%)	2.41e-10	Metabolism	Global and overview maps
Gastric acid secretion	ko04971	22 (3.29%)	1.51e-09	Organismal Systems	Digestive system
Oxytocin signaling pathway	ko04921	28 (4.19%)	1.71e-09	Organismal Systems	Endocrine system
Citrate cycle (TCA cycle)	ko00020	17 (2.54%)	2.21e-09	Metabolism	Carbohydrate metabolism
Calcium signaling pathway	ko04020	25 (3.74%)	2.86e-06	Environmental Information Processing	Signal transduction
MAPK signaling pathway	ko04010	29 (4.34%)	2.82e-05	Environmental Information Processing	Signal transduction
Dopaminergic synapse	ko04728	19 (2.84%)	0.000151	Organismal Systems	Nervous system
Leukocyte transendothelial migration	ko04670	28 (4.19%)	0.000163	Organismal Systems	Immune system
Folate biosynthesis	ko00790	9 (1.35%)	0.000292	Metabolism	Metabolism of cofactors and vitamins
Biosynthesis of amino acids	ko01230	14 (2.1%)	0.00158	Metabolism	Global and overview maps
Biosynthesis of antibiotics	ko01130	3 (4.64%)	0.00213	Metabolism	Global and overview maps
Alanine, aspartate and glutamate metabolism	ko00250	8 (1.2%)	0.00281	Metabolism	Amino acid metabolism
Metabolism of xenobiotics by cytochrome P450	ko00980	13 (1.95%)	0.00401	Metabolism	Xenobiotics biodegradation and metabolism
Aminobenzoate degradation	ko00627	9 (1.35%)	0.00505	Metabolism	Xenobiotics biodegradation and metabolism
Drug metabolism - cytochrome P450	ko00982	13 (1.95%)	0.00561	Metabolism	Xenobiotics biodegradation and metabolism
cAMP signaling pathway	ko04024	21 (3.14%)	0.00676	Environmental Information Processing	Signal transduction
Glycolysis/Gluconeogenesis	ko00010	11 (1.65%)	0.0107	Metabolism	Carbohydrate metabolism
Protein digestion and absorption	ko04974	21 (3.14%)	0.0311	Organismal Systems	Digestive system
Cysteine and methionine metabolism	ko00270	8 (1.2%)	0.0327	Metabolism	Amino acid metabolism
Amino sugar and nucleotide sugar metabolism	ko00520	12 (1.8%)	0.0412	Metabolism	Carbohydrate metabolism

The Bonferroni correction method was used for multiple hypothesis test correction and FDR-corrected *P* < 0.05 as cut-off

**Table 2:** Selected KEGG pathways significantly enriched for down-regulated genes in sugar-only diet (SD) versus normal diet (ND)

KEGG pathway	Pathway ID	DEGs (1229)	*P*-value	Level 1	Level 2
Cell cycle	ko04110	49 (3.99%)	9.25e-19	Cellular Processes	Cellular Processes
DNA replication	ko03030	28 (2.28%)	1.66e-16	Genetic Information Processing	Replication and repair
Cell cycle - yeast	ko04111	39 (3.17%)	4.57e-13	Cellular Processes	Cell growth and death
Meiosis - yeast	ko04113	28 (2.28%)	1.22e-08	Cellular Processes	Cell growth and death
Mismatch repair	ko03430	14 (1.14%)	3.69e-07	Genetic Information Processing	Replication and repair
RNA transport	ko03013	51 (4.15%)	5.08e-07	Genetic Information Processing	Translation
Nucleotide excision repair	ko03420	19 (1.55%)	2.27e-06	Genetic Information Processing	Replication and repair
Ubiquitin mediated proteolysis	ko04120	36 (2.93%)	7.68e-06	Genetic Information Processing	Folding, sorting and degradation
Progesterone-mediated oocyte maturation	ko04914	24 (1.95%)	1.55e-05	Organismal Systems	Endocrine system
Fanconi anemia pathway	ko03460	14 (1.14%)	0.000144	Genetic Information Processing	Replication and repair
Protein processing in endoplasmic reticulum	ko04141	34 (2.77%)	0.00165	Genetic Information Processing	Folding, sorting and degradation
Spliceosome	ko03040	35 (2.85%)	0.00193	Genetic Information Processing	Transcription
mTOR signaling pathway	ko04150	5 (0.41%)	0.00423	Environmental Information Processing	Signal transduction
mRNA surveillance pathway	ko03015	22 (1.79%)	0.0101	Genetic Information Processing	Translation
p53 signaling pathway	ko04115	12 (0.98%)	0.0124	Cellular Processes	Cell growth and death
Oocyte meiosis	ko04114	23 (1.87%)	0.0131	Cellular Processes	Cell growth and death
Insulin signaling pathway	ko04910	18 (1.4%)	0.0154	Organismal Systems	Endocrine system
RNA degradation	ko03018	19 (1.55%)	0.03263363	Genetic Information Processing	Folding, sorting and degradation
ErbB signaling pathway	ko04012	15 (1.22%)	0.03373181	Environmental Information Processing	Signal transduction
Insect hormone biosynthesis	ko00981	11 (0.9%)	0.03590656	Metabolism	Metabolism of terpenoids and polyketides

The Bonferroni correction method was used for multiple hypothesis test correction and FDR-corrected *P* < 0.05 as cut-off

### Ovary development

**Fig. 3:**
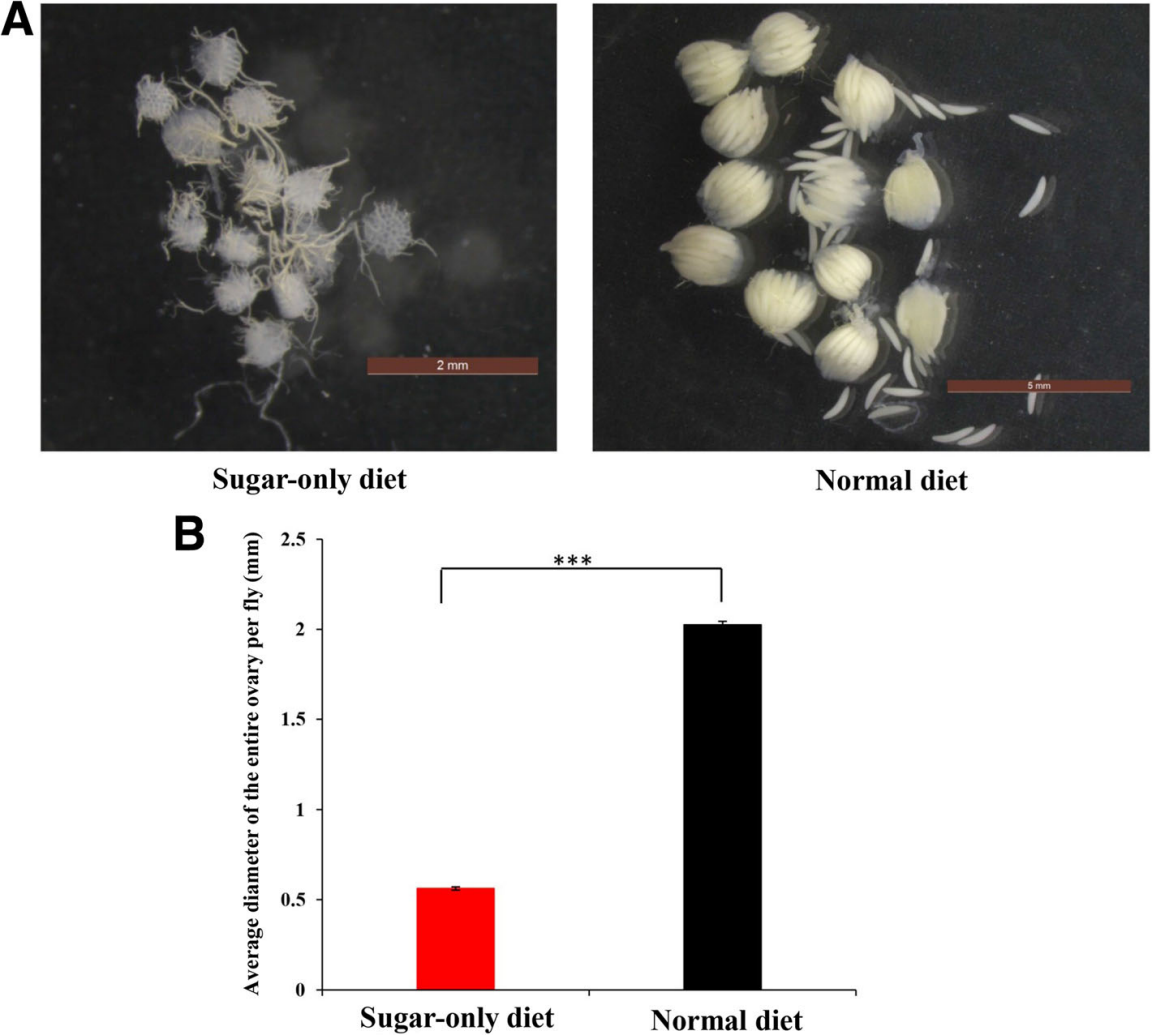
**a** Ovaries of 30-day-old females fed on sugar-only or normal diet, respectively. **b** The difference in the size of ovaries from the sugar-only and normal diet groups is indicated by the average of maximum diameter. Statistically significant differences between diet and control are indicated with *** (at *P* < 0.001, *t*-test)

The ovary size of the female adults reared with the ND was significantly greater than those of females reared with SD (*P <* 0.001) (Fig. 3a). The average ovary size of the ND group was 2.04 ± 0.01 mm, whereas the average ovary size for the SD group was 0.61 ± 0.003 mm (Fig. 3b).

### Starvation and desiccation treatments

**Fig. 4:**
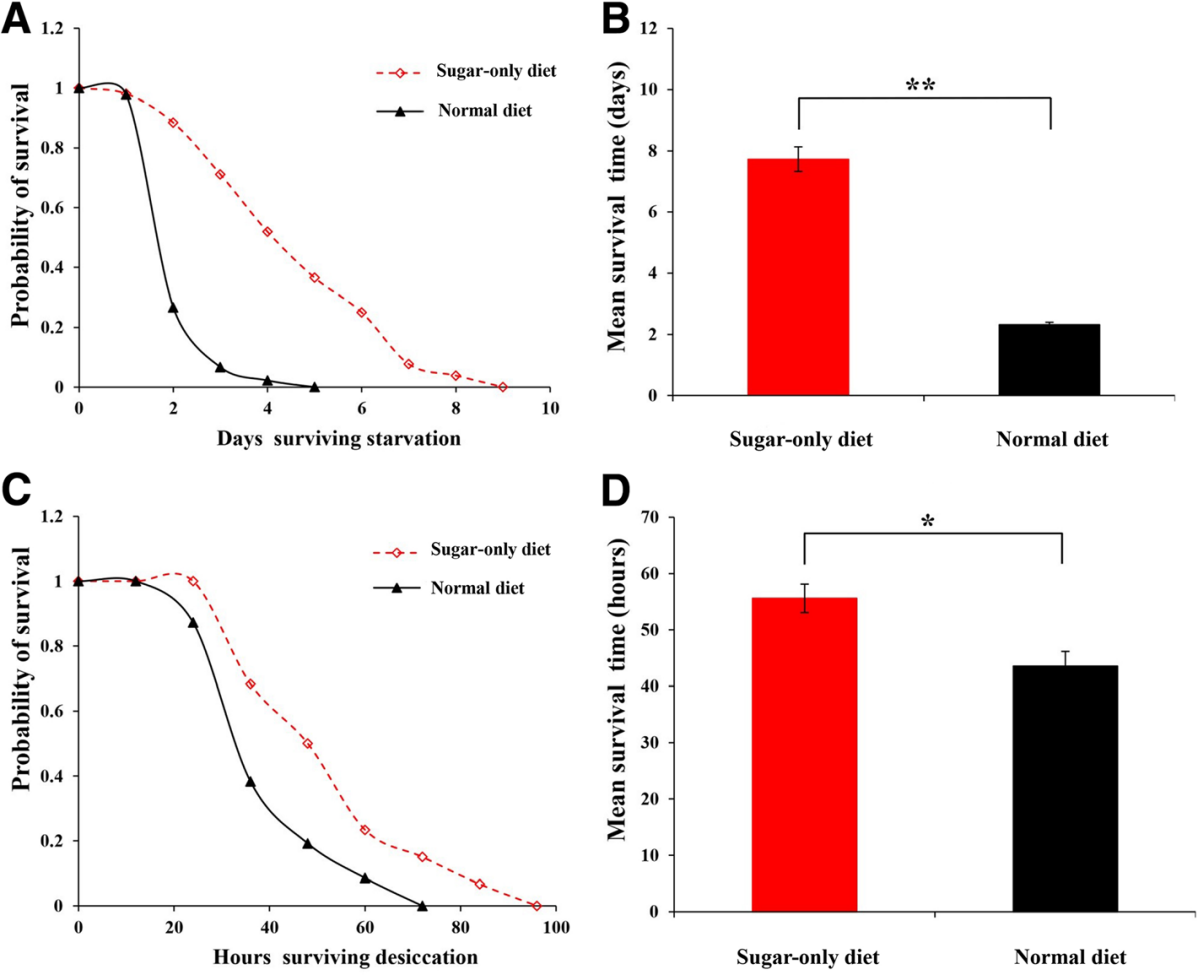
Survival curves for starvation resistance (**a**) and desiccation resistance (**c**), and mean time before death from starvation stress (**b**) and desiccation stress (**d**) in *Bactrocera dorsalis* derived from either sugar-only diet or normal regimes. Statistical difference was determined by the independent samples *t*-test (* *P* < 0.05, ** *P* < 0.01)

Starvation resistance was significantly affected by the quality of the diets (Fig. 4a-b), and *B. dorsalis* fed on an SD had longer survival time than those on the ND, and the mean survival time was 4.82 ± 0.49 and 2.21 ± 0.06 days, respectively (*P* = 0.006). In terms of the desiccation treatment, the mean survival time was 55.60 ± 2.53 and 43.57 ± 2.53 h under the SD and ND diets, respectively, which suggested that SD significantly increased the desiccation resistance (*P* = 0.01) (Fig. 4c, d).

### Lifespan

**Fig. 5:**
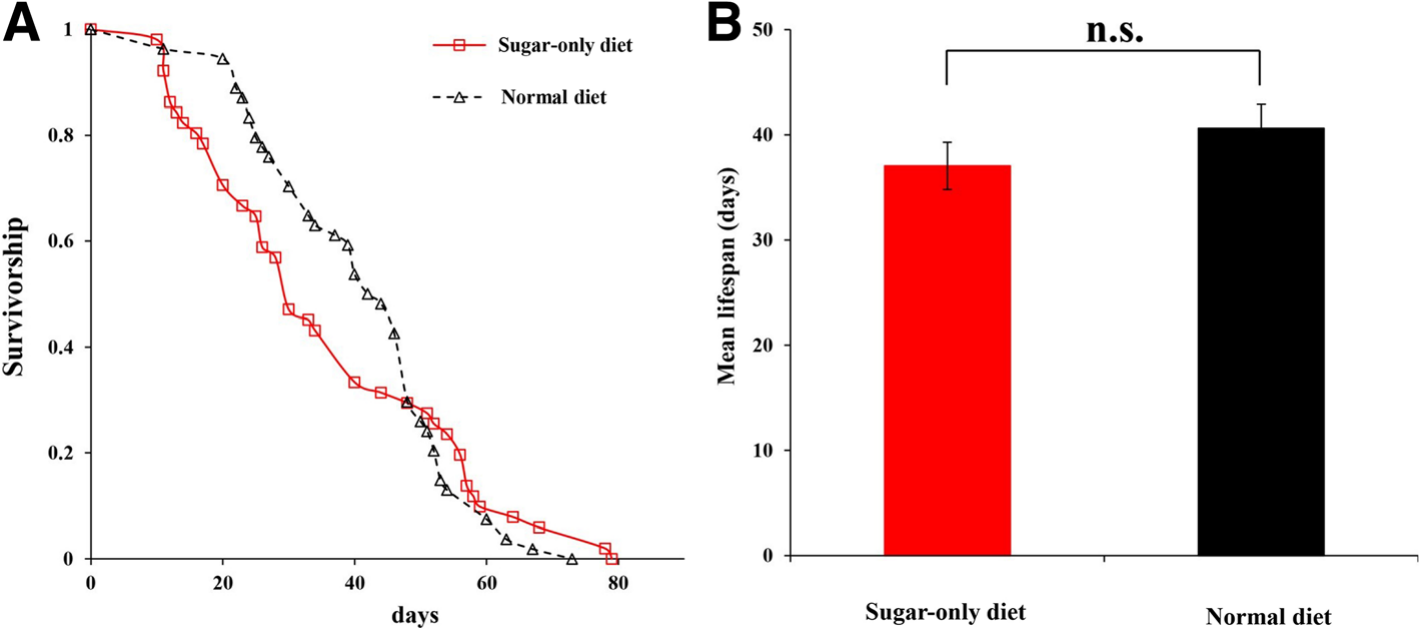
**a** Survival curves of female *Bactrocera dorsalis* adults fed on sugar-only and normal diet. **b** Mean lifespan of *Bactrocera dorsalis* adults fed on sugar-only and normal diet. Statistical difference was determined by the independent samples *t*-test (n.s., not significant)

No significant effect on lifespan was observed using different diets (*P* = 0.27) (Fig. 5a). The mean lifespan was 37.05 ± 2.25 days and 40.64 ± 2.24 days under the SD and ND diets, respectively (Fig. 5b). These results suggested that SD repressed ovary development without extending the lifespan of *B. dorsalis*.

### Antioxidant responses

**Fig. 6:**
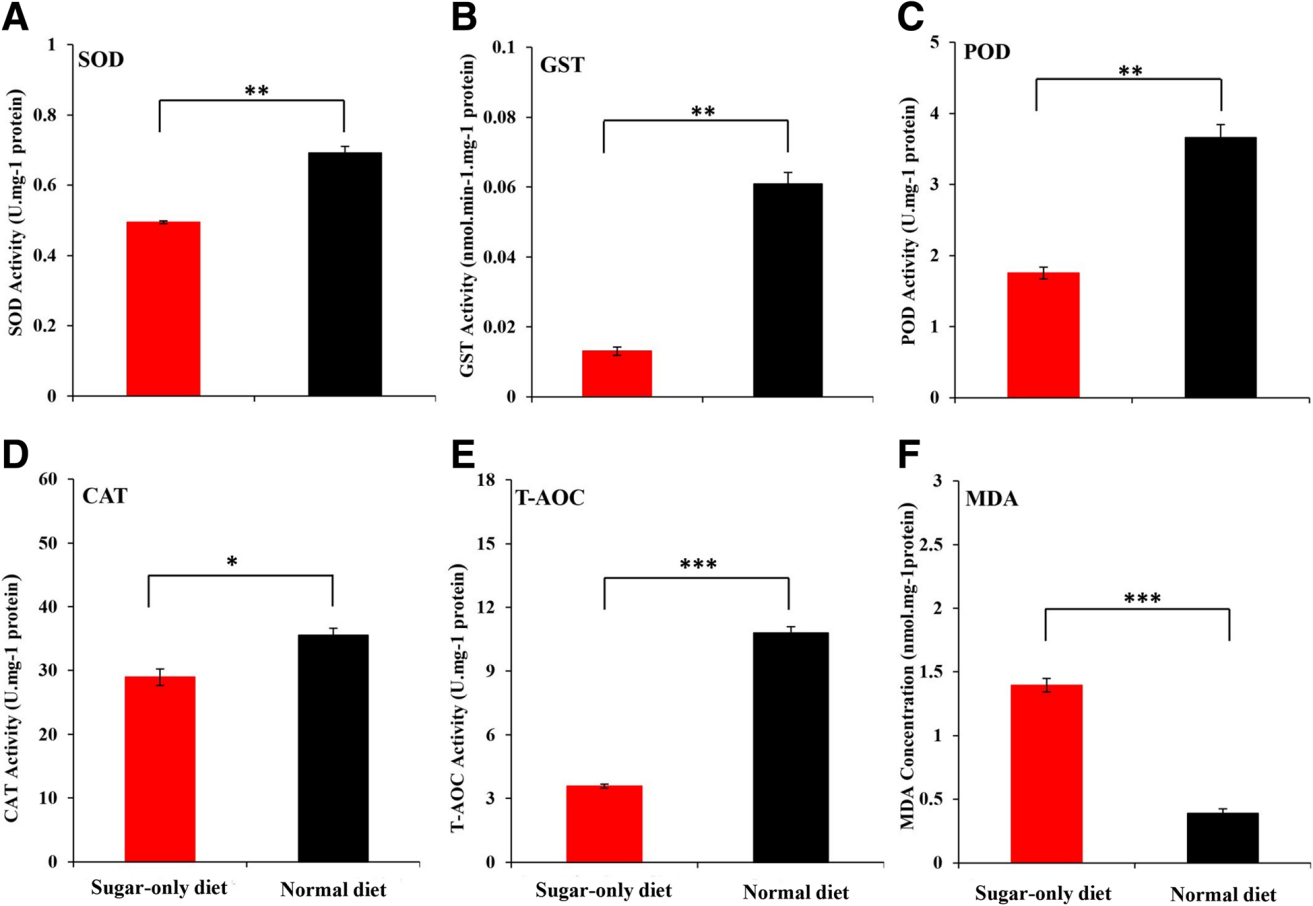
Antioxidant responses and lipid peroxidation assay of *Bactrocera dorsalis* fed on sugar-only and normal diet for 30 days. Each value represents the mean ± SE of three replicates. **a** SOD activity; **b** GST activity; **c** POD activity; **d** CAT activity; **e** total antioxidant capacity (T-AOC); and **f** malondialdehyde (MDA) concentration. Statistical difference was determined by the independent samples *t*-test (* *P* < 0.05, ** *P* < 0.01, *** *P* < 0.001)

The antioxidant enzyme activities of *B. dorsalis* fed on the SD and ND for 30 days significantly differed (Fig. 6a-d). Flies fed on the SD showed lower SOD (*P* = 0.007), GST (*P* = 0.002), POD (*P* = 0.003), and CAT (*P* = 0.02) activities than those on ND. Compared to the ND diet, *B. dorsalis* exhibited lower T-AOC when fed on the SD diet (*P* < 0.001) (Fig. 6e). Flies fed on the SD had higher MDA concentrations (*P* < 0.001), suggesting that they had higher lipid peroxidation levels (Fig. 6f).

## Discussion

In the present study, we used RNA-seq to identify genes that were regulated by nutrient signals in *B. dorsalis*. Similar to *Drosophila* [14], we observed that *B. dorsalis* elicited a strong response to SD as revealed by changes in gene expression levels (Additional file 4: Figure S2), and a large number of DEGs were significantly enriched in metabolic pathways (Additional file 5: Figure S3). These changes may have led to the alterations in the storage and metabolism of fats and carbohydrates [21, 35], as well as induction of autophagy [36], which in turn resulted in the growth and developmental arrest of *B. dorsalis*, as well as maintenance of homeostasis under poor nutrient conditions [37]. We also observed an inhibition of ‘cell growth and death’ pathways (Table 2), suggesting that an SD prevent cell growth and cell cycle progression in *B. dorsalis* [38]. Three pathways that are associated with transcriptional activities were downregulated under an SD regimen, including: ‘RNA degradation’, ‘spliceosome’ and ‘mRNA surveillance’ (Table 2), which could affect the fidelity and quality of mRNA molecules [39]. Calcium signaling pathway was activated in response to the SD (Table 1), and a previous study has reported that calcium and calmodulin are key components of signal transduction pathways that may be involved in stress responses to various environmental stressors [39]. Similar to a previous report that showed that the nutritional environment of insects influences the immune status of *D. melanogaster*, the ‘immune system’ category was also significantly enriched with 28 upregulated genes under the SD regime (Table 1), which suggests that the chronic nutritional stress affects adult immunity by influencing plastic allocation of resources [40]. Another interesting finding is that genes involved in the folate biosynthesis pathway were upregulated (Table 1); these have been implicated in the aging process in various organisms [41, 42]. In particular, folate is a precursor to methionine, which is then converted into s-adenosyl methionine (SAM), a cell-wide donor of methyl groups. It is through this pathway that folate levels have been associated with DNA methylation levels and aging [43]. Protein starvation disrupts amino acid and fatty acid metabolism in *Drosophila* [38]. Similarly, the ‘biosynthesis of the amino acids alanine’, ‘aspartate and glutamate metabolism’, and ‘Cysteine and methionine metabolism’ pathways were also significantly enriched in our study. The complete lack of yeast in the diet would certainly disrupt amino acid and protein biosynthesis and metabolism, and the flies responded to this stress by increasing their rate for amino acids metabolism.

Additionally, the current study found that 66 upregulated genes were significantly enriched in the ‘oxidative phosphorylation’ pathway, which have been reported to play a central role in eukaryotic metabolism to provide the energy (ATP) that is required for insect survival [44]. The overexpression of genes that are involved in energy production in *B. dorsalis* might reflect the costs of survival in the chronic SD conditions. Interestingly, as one of the major pathways associated with lifespan, the oxidative phosphorylation pathway is also activated in *D. melanogaster* under protein restricted diet [45]. Furthermore, 112 (16.0%) downregulated genes were enriched in the ‘reproductive process’ GO terms (Additional file 7: Table S5). Here, the upregulation of pathways involved in energy production and the downregulation of pathways involved in reproduction suggest a trade-off between reproduction versus somatic maintenance (stress resistance) and lifespan [46]. Similarly, in most insects reproduction trades off with somatic maintenance and lifespan exists, and it has been documented at the phenotypic, physiological, or quantitative genetic level in numerous species and represents a major constraint upon physiology and the evolution of life histories [47, 48]. At the physiological level, the possible explanation of this trade-off is that the energetically costly process of reproduction ‘competes’ with the energy demands of somatic maintenance and survival [49]. At the same time, *Drosophila* and other solitary insects have showed that this trade-off is achieved by an endocrine network that integrates insulin signaling pathway, JH, and the yolk precursor vitellogenin (Vg) [50]. Although, the extended lifespan is often associated with reduced fecundity, recent studies have also shown that fecundity and lifespan have different nutritional optima, and that there is no concomitant trade-off, indicating a controversial area [51].

As reported, the negative effect of InR and insulin receptor substrate (IRS) RNAi-mediated silencing on reproductive events has been observed in several insect species, thereby suggesting the involvement of the insulin pathway in the control of reproductive system development [52, 53]. Two recent studies have also reported that the nutritional environment affects the hormone production in insects, and knocking down the expression of genes involved in JH biosynthesis (JH acid methyltransferase) blocks Vg synthesis and disrupts ovarian development [54, 55]. A similar negative effect on JH biosynthesis and adult Vg has also been obtained by means of InR RNAi in the penultimate and last instar nymphs of the *Blattella germanica* [53]. Insulin signaling pathway affects JH production, which is the central regulator of fecundity, thereby regulating the synthesis of Vg [56]. As precursor proteins of vitellin, the major yolk egg protein, Vg is directly involved in reproduction. Downregulation of this network, for example in response to low nutrient availability or other environmental changes (temperature or photoperiod), promotes maintenance and survival at the expense of reproduction [57].

In the present study, we found that SD downregulates the nutritional sensor of the TOR (Additional file 8: Figure S4), insulin (Fig. 2a), and JH biosynthesis (Fig. 2b) signaling pathways in *B. dorsalis*. The TOR, which is a serine/threonine kinase, is highly conserved in most eukaryotes and plays a key role in the transduction of nutritional signals. The direct inhibition of TOR signaling pathway has been shown to extend the lifespan of yeast [58], flies [59], and mammals [60]. Here, among the down-regulated genes, *Vg1* (gene ID: 105,232,170; diverge probability = 0.99) and *Vg2* (gene ID: 105,222,970; diverge probability = 0.99) had more than 100-fold lower expression under the SD diet in *B. dorsalis* (Additional file 9: Table S3). Therefore, these results suggested that insulin signaling pathway asserts its nutritionally linked influence on Vg expression of *B. dorsalis* by contributing to the control of biosynthesis and secretion of JH under the SD regimen [61].

As expected, ovary development of *B. dorsalis* under the SD regimen was completely blocked, and no mature eggs were observed (Fig. 3), which implied that dietary protein plays an important role in the regulation of ovarian development in *B. dorsalis* [62]. Similar to *D. melanogaster*, the desiccation resistance has a positive relationship with starvation resistance, and the increasing levels of starvation resistance were also more resistant to desiccation [63]. High sugar diets are often associated with higher triglyceride levels and therefore, have better starvation resistance [64]. In the present study, SD induced an increase in starvation and desiccation resistance in *B. dorsalis*, thereby suggesting a trade-off between stress resistance and reproduction under the SD diet. According to the several aging theories, the extended lifespan is often correlated with increased resistance against various stressors, and several studies have shown genetic correlations among resistance to several stresses and longevity, thereby strongly suggesting that genetic basis of lifespan and stress resistance overlap [65–67].

However, our study found that the downregulated Insulin-JH-Vg signaling network, decreased reproduction and increased stress resistance were not associated with the lifespan extension under the SD regimen in *B. dorsalis*. Antioxidant activity and the accumulation of oxidative damage caused by ROS are closely related to lifespan in various organisms [17]. ROS are formed by the incomplete reduction of oxygen, primarily in the mitochondria during aerobic metabolism, and antioxidant systems were usually able to keep this oxidative stress under control [68]. When ROS production exceeds antioxidant defenses, cells enter a pro-oxidant state called oxidative stress and damage occurs. When this damage is not repaired, damages accumulate in cells, which in turn results in further oxidative damage, thereby causing aging and ultimately, death [69, 70]. Moreover, a study involving *Teleogryllus commodus* has shown that dietary manipulation have significant effects on oxidative damage and antioxidant protection [71]. Intriguingly, our RNA-seq data showed that the antioxidant gene expressions of SOD (gene ID: 105,232,170; diverge probability = 0.90), GST (gene ID: 105,229,686; diverge probability = 0.96), and CAT (gene ID: 105,222,970; diverge probability = 0.87) were downregulated under the SD diet (Additional file 9: Table S3). Meanwhile, in agreement with our RNA-seq data, the present study showed that the SD decreased antioxidant enzyme activities (of SOD, GST, POD, and CAT) and antioxidant capacity (T-AOC) in *B. dorsalis*. As a major oxidation product of peroxidized polyunsaturated fatty acids, MDA can be used as a biological marker for oxidative damage. Here, we also found that SD increased the MDA concentrations of *B. dorsalis* compared to ND regime (Fig. 6). These results indicated that antioxidants of *B. dorsalis* cannot keep up with the increase in oxidative damage that was induced by SD diets. Hence, we believe that the flies did not extend the lifespan amidst oxidative damage accumulation and under the unnatural and extreme diets (SD). Similarly, the relationship between sugar-only diet and lifespan have also been studied in *Anastrepha ludens* and *Drosophila*, which showed less fecundity without lifespan extension under SD regimen [11, 72].

## Conclusion

Our data show that SD leads to widespread changes in the expression levels of genes involved in energy production, reproduction, as well as the nutritional sensor of the TOR, insulin, and JH biosynthesis signaling pathways in *B. dorsalis*. The SD also significantly affected the phenotypes and antioxidant responses, including an increase in stress resistance, disruption of ovary development, decrease in antioxidant enzyme activities, and accumulation of oxidative damage. Furthermore, yeast provides protein, micronutrients, and salt, although which component or combination of components plays the major role remains unclear. We anticipate conducting another study that will focus on elucidating this mechanism.

## Additional files

Additional file 1: Table S1. Data output quality and mapping rates for the examined samples of *Bactrocera dorsalis*. (DOCX 17 kb)

Additional file 2: Table S2. The items of quality control for each sample of *Bactrocera dorsalis*. (DOCX 17 kb)

Additional file 3: Figure S1. Histogram distribution of gene expression levels of each sample. X-axis is FPKM value (the coordinate has been changed by logarithm for better view). Y-axis is gene number of corresponding FPKM. A: ND-1, ND-2 and ND-3; B: SD-1, SD-2 and SD-3. (JPEG 565 kb)

Additional file 4: Figure S2. Analysis of DEGs between the two diets. The DEGs were defined as abs (log2(Y/X) ≥ 1 and diverge probability ≥0.8). (JPEG 621 kb)

Additional file 5: Figure S3. GO and KEGG classification on DEGs for sugar-only diet and normal diet. (A) GO terms for DEGs. (B) KEGG pathway for DEGs. The Bonferroni correction method was used for multiple hypothesis test correction and FDR-corrected *P* < 0.05 as cut-off. (JPEG 1050 kb)

Additional file 6: Table S4. Selected GO terms significantly enriched for upregulated genes in SD versus ND. (DOCX 19 kb)

Additional file 7: Table S5. Selected GO terms significantly enriched for downregulated genes in SD versus ND. (DOCX 20 kb)

Additional file 8: Figure S4. Downregulated genes involved in the TOR signaling pathway in response to the sugar-only diet. The Bonferroni correction method was used for multiple hypothesis test correction and FDR-corrected *P* < 0.05 as cut-off. (JPEG 412 kb)

Additional file 9: Table S3. Detailed information on the DEGs of chronic SD and ND diets in *Bactrocera dorsalis*. (XLSX 556 kb)

## Abbreviations

ATP: Adenosine triphosphate
CAMP: Cyclic adenosine 3′,5′-monophosphate
CAT: Catalase
CDNB: 1-chloro-2,4-dinitrobenzene
DEGs: Different expression genes
FPKM: Fragment per kilobase of exon model per million mapped reads
GO: Gene Ontology
GSH: Glutathione
GST: Glutathione S-transferase
HISAT: Hierarchical indexing for spliced alignment of transcripts
IGF: Insulin-like growth factors
ILPs: Insulin-like peptides
InR: Insulin receptors
IRS: Insulin receptor substrate
JH: Juvenile hormone
KEGG: Kyoto encyclopedia of genes and genomes
LPO: Lipid peroxidation
MAPK: Mitogen-activated protein kinase
MDA: Malondialdehyde
ND: Normal diet
PI3K: PI3 kinase
POD: Peroxidase
ROS: Reactive oxygen species
RSEM: RNA-Seq by expectation maximization
SAM: S-adenosyl methionine
SD: Sugar-only diet
SOD: Superoxide dismutase
T-AOC: Total antioxidant capacity
TOR: Target of rapamycin
Vg: Vitellogenin
