# Supplementary material for: Phenotypes, antioxidant responses, and gene expression changes accompanying a sugar-only diet in Bactrocera dorsalis (Hendel) (Diptera: Tephritidae)
Source: BMC Evol Biol. 2017 Aug 17;17:194. doi: 10.1186/s12862-017-1045-5 (PMC5559826; doi:10.1186/s12862-017-1045-5)
Supplement: Supplementary file 1 — Data output quality and mapping rates for the examined samples of Bactrocera dorsalis. (DOCX 17 kb) [file 12862_2017_1045_MOESM1_ESM.docx]

**Additional file 1: Table S1** Data output quality and mapping rates for the examined samples of *Bactrocera dorsalis*

| **Samples** | **Raw**  **Data Size (bp)** | **Raw**  **Reads Number** | **Clean**  **Data Size (bp)** | **Clean**  **Reads Number** | **Clean**  **Data Rate (%)** | **Length of**  **Sequence Reads**  **(bp)** | **GC**  **(%)** |
| --- | --- | --- | --- | --- | --- | --- | --- |
| ND-1 | 1,197,866,850 | 23,957,337 | 1,197,537,150 | 23,950,743 | 99.97 | 50 | 43.32 |
| ND-2 | 1,197,875,250 | 23,957,505 | 1,197,456,000 | 23,949,120 | 99.96 | 50 | 43.43 |
| ND-3 | 1,197,879,250 | 23,957,585 | 1,197,738,000 | 23,954,760 | 99.98 | 50 | 43.02 |
| SD-1 | 1,197,869,900 | 23,957,398 | 1,197,661,350 | 23,953,227 | 99.98 | 50 | 43.61 |
| SD-2 | 1,197,857,850 | 23,957,157 | 1,197,689,400 | 23,953,788 | 99.98 | 50 | 42.76 |
| SD-3 | 1,197,865,850 | 23,957,317 | 1,197,685,750 | 23,953,715 | 99.98 | 50 | 43.47 |

Clean Data Rate (%) = Clean Reads Number / Raw Reads Number.
