# Supplementary material for: Phenotypes, antioxidant responses, and gene expression changes accompanying a sugar-only diet in Bactrocera dorsalis (Hendel) (Diptera: Tephritidae)
Source: BMC Evol Biol. 2017 Aug 17;17:194. doi: 10.1186/s12862-017-1045-5 (PMC5559826; doi:10.1186/s12862-017-1045-5)
Supplement: Supplementary file 2 — The items of quality control for each sample of Bactrocera dorsalis. (DOCX 17 kb) [file 12862_2017_1045_MOESM2_ESM.docx]

**Additional file 2: Table S2** Quality control items for each sample of *Bactrocera dorsalis*

| **Samples** | **Clean Read**  **Q20 (%) ≥ 90** | **Clean Reads**  **≥ 20 (M)** | **Gene Unique Mapping**  **Ratio (%) ≥ 80** | **Genome Mapping**  **Ratio (%) ≥ 50** |
| --- | --- | --- | --- | --- |
| ND-1 | 94.1 (Y) | 23.95 (Y) | 98.30 (Y) | 85.96 (Y) |
| ND-2 | 93.3 (Y) | 23.95 (Y) | 98.61 (Y) | 84.15 (Y) |
| ND-3 | 93.1 (Y) | 23.95 (Y) | 98.47 (Y) | 86.04 (Y) |
| SD-1 | 93.4 (Y) | 23.95 (Y) | 98.01 (Y) | 81.21 (Y) |
| SD-2 | 93.5 (Y) | 23.95 (Y) | 98.06 (Y) | 80.23 (Y) |
| SD-3 | 92.6 (Y) | 23.95 (Y) | 98.35 (Y) | 81.75 (Y) |

'Y' means sample passed this QC item and 'N' means failed.
