# Supplementary figures and images for: Phenotypes, antioxidant responses, and gene expression changes accompanying a sugar-only diet in Bactrocera dorsalis (Hendel) (Diptera: Tephritidae)
Source: BMC Evol Biol. 2017 Aug 17;17:194. doi: 10.1186/s12862-017-1045-5 (PMC5559826; doi:10.1186/s12862-017-1045-5)

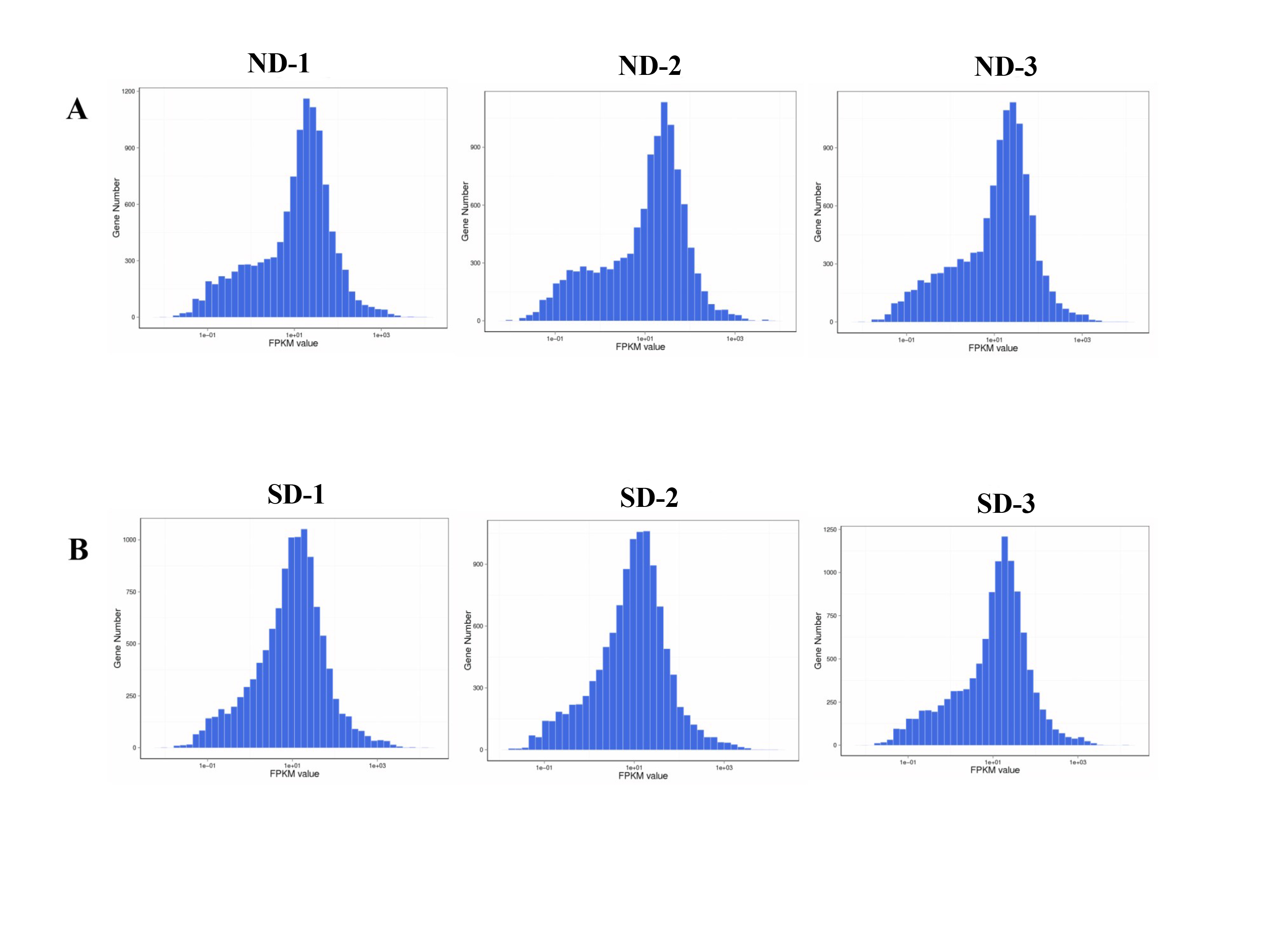

Supplement: Supplementary file 3 — Histogram distribution of gene expression levels of each sample. X-axis is FPKM value (the coordinate has been changed by logarithm for better view). Y-axis is gene number of corresponding FPKM. A: ND-1, ND-2 and ND-3; B: SD-1, SD-2 and SD-3. (JPEG 565 kb) [file 12862_2017_1045_MOESM3_ESM.jpg]

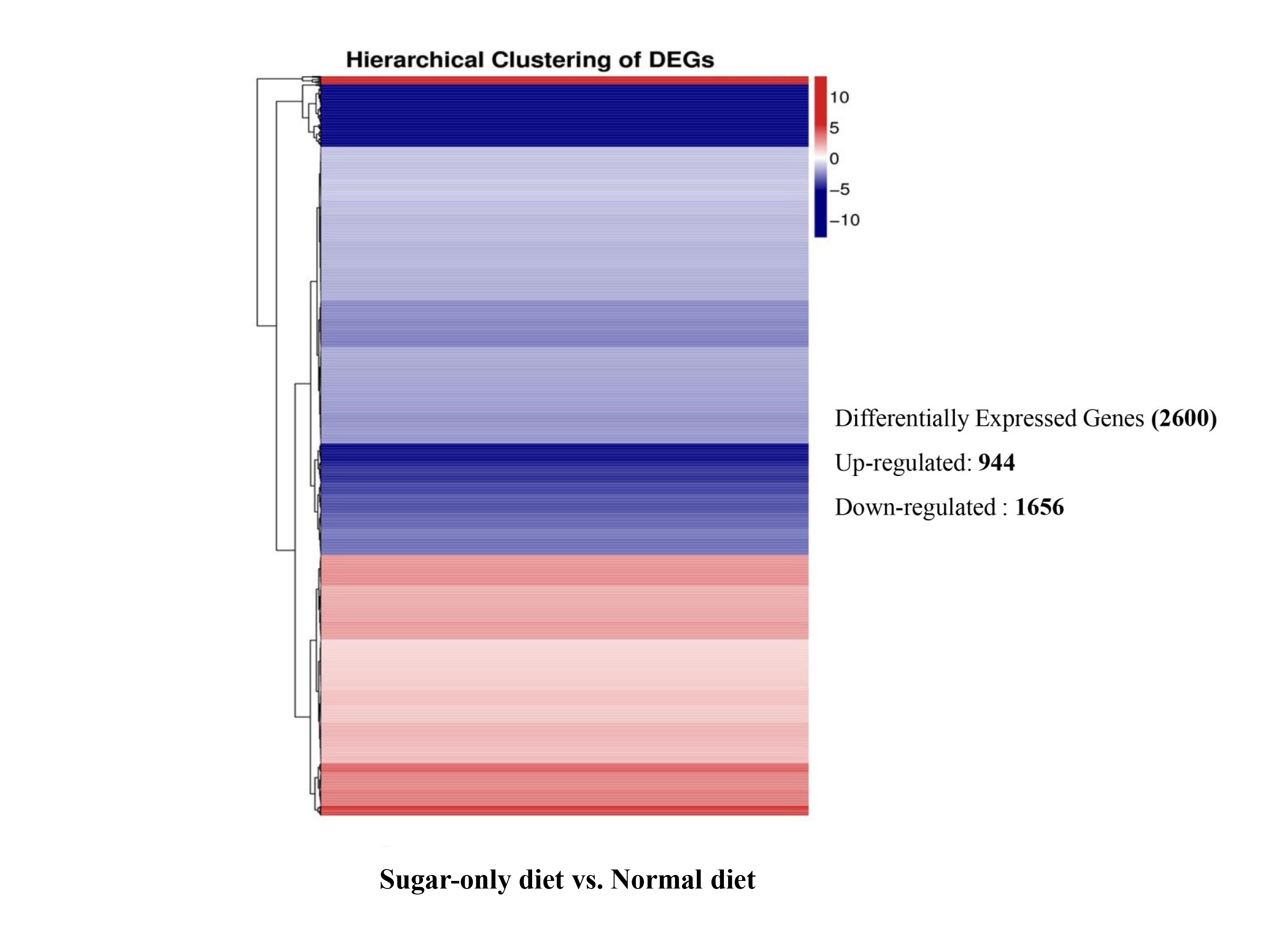

Supplement: Supplementary file 4 — Analysis of DEGs between the two diets. The DEGs were defined as abs (log2(Y/X) ≥ 1 and diverge probability ≥0.8). (JPEG 621 kb) [file 12862_2017_1045_MOESM4_ESM.jpg]

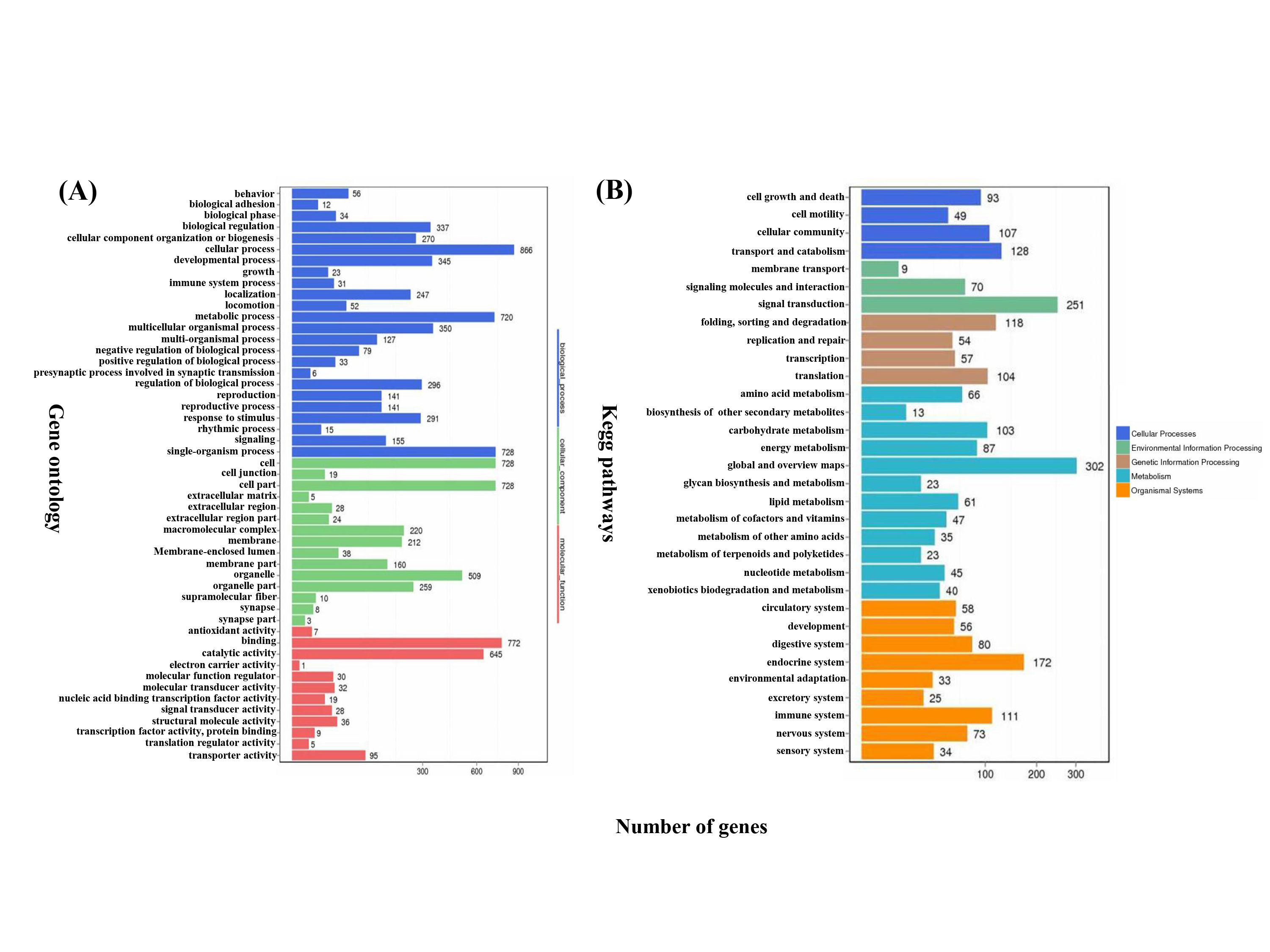

Supplement: Supplementary file 5 — GO and KEGG classification on DEGs for sugar-only diet and normal diet. (A) GO terms for DEGs. (B) KEGG pathway for DEGs. The Bonferroni correction method was used for multiple hypothesis test correction and FDR-corrected P < 0.05 as cut-off. (JPEG 1050 kb) [file 12862_2017_1045_MOESM5_ESM.jpg]

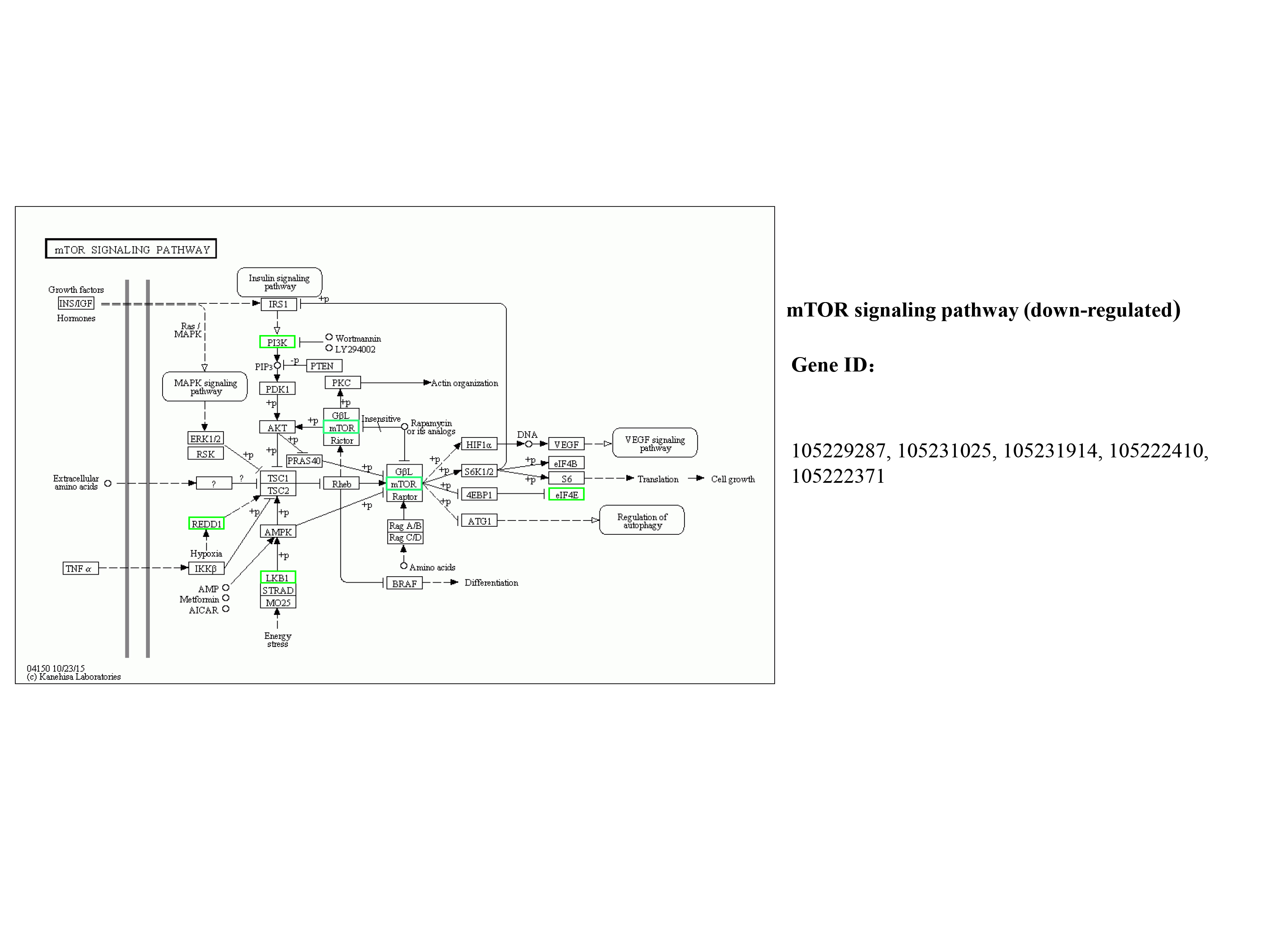

Supplement: Supplementary file 8 — Downregulated genes involved in the TOR signaling pathway in response to the sugar-only diet. The Bonferroni correction method was used for multiple hypothesis test correction and FDR-corrected P < 0.05 as cut-off. (JPEG 412 kb) [file 12862_2017_1045_MOESM8_ESM.jpg]
