# Supplementary material for: Phenotypes, antioxidant responses, and gene expression changes accompanying a sugar-only diet in Bactrocera dorsalis (Hendel) (Diptera: Tephritidae)
Source: BMC Evol Biol. 2017 Aug 17;17:194. doi: 10.1186/s12862-017-1045-5 (PMC5559826; doi:10.1186/s12862-017-1045-5)
Supplement: Supplementary file 6 — Selected GO terms significantly enriched for upregulated genes in SD versus ND. (DOCX 19 kb) [file 12862_2017_1045_MOESM6_ESM.docx]

**Additional file 6: Table S4** Selected GO terms significantly enriched for up-regulated genes in sugar-only diet (SD) versus normal diet (ND)

| **GO term** | **GO ID** | **DEGs (447)** | **FDR-corrected**  **(*P-value*)** |
| --- | --- | --- | --- |
| generation of precursor metabolites and energy | GO:0006091 | 48 (10.7%) | 1.15e-23 |
| cellular respiration | GO:0045333 | 43 (9.6%) | 2.14e-23 |
| energy derivation by oxidation of organic compounds | GO:0015980 | 44 (9.8%) | 5.54e-23 |
| oxidation-reduction process | GO:0055114 | 44 (9.8%) | 4.16e-22 |
| respiratory electron transport chain | GO:0022904 | 32 (7.2%) | 1.89e-21 |
| electron transport chain | GO:0022900 | 32 (7.2%) | 4.53e-20 |
| purine nucleoside triphosphate biosynthetic process | GO:0009145 | 19 (4.3%) | 3.53e-09 |
| purine ribonucleoside triphosphate biosynthetic process | GO:0009206 | 19 (4.3%) | 3.53e-09 |
| nucleoside triphosphate biosynthetic process | GO:0009142 | 19 (4.3%) | 1.38e-08 |
| ribonucleoside triphosphate biosynthetic process | GO:0009201 | 19 (4.3%) | 1.38e-08 |
| ATP biosynthetic process | GO:0006754 | 13 (2.9%) | 9.23e-08 |
| purine nucleoside biosynthetic process | GO:0042451 | 13 (2.9%) | 9.23e-08 |
| ATP metabolic process | GO:0046034 | 13 (2.9%) | 9.23e-08 |
| purine ribonucleoside biosynthetic process | GO:0046129 | 13 (2.9%) | 9.23e-08 |
| actomyosin structure organization | GO:0031032 | 14 (3.1%) | 2.32e-07 |
| nucleoside biosynthetic process | GO:0009163 | 13 (2.9%) | 2.68e-07 |
| purine nucleoside metabolic process | GO:0042278 | 13 (2.9%) | 2.68e-07 |
| ribonucleoside biosynthetic process | GO:0042455 | 13 (2.9%) | 2.68e-07 |
| purine ribonucleoside metabolic process | GO:0046128 | 13 (2.9%) | 2.68e-07 |
| glycosyl compound biosynthetic process | GO:1901659 | 13 (2.9%) | 2.68e-07 |
| nucleoside metabolic process | GO:0009116 | 13 (2.9%) | 3.83e-06 |
| ribonucleoside metabolic process | GO:0009119 | 13 (2.9%) | 3.83e-06 |
| purine ribonucleotide metabolic process | GO:0009150 | 13 (2.9%) | 3.83e-06 |
| ribose phosphate metabolic process | GO:0019693 | 13 (2.9%) | 1.62e-05 |
| hydrogen transport | GO:0006818 | 15 (3.4%) | 1.66e-05 |
| single-organism metabolic process | GO:0044710 | 120 (26.8%) | 5.65e-05 |
| purine nucleoside triphosphate metabolic process | GO:0009144 | 33 (7.4%) | 0.00011 |
| ribonucleoside triphosphate metabolic process | GO:0009199 | 33 (7.4%) | 0.00016 |
| single-organism biosynthetic process | GO:0044711 | 33 (7.4%) | 0.00117 |
| carbohydrate derivative metabolic process | GO:1901135 | 27 (6.0%) | 0.00512 |
| muscle structure development | GO:0061061 | 17 (3.8%) | 0.00566 |
| somatic muscle development | GO:0007525 | 7 (1.6%) | 0.00879 |
| purine nucleotide biosynthetic process | GO:0006164 | 14 (3.1%) | 0.00976 |
| purine nucleotide metabolic process | GO:0006163 | 15 (3.4%) | 0.01109 |
| nucleoside phosphate biosynthetic process | GO:1901293 | 20 (4.5%) | 0.01548 |
| purine-containing compound biosynthetic process | GO:0072522 | 14 (3.1%) | 0.01759 |
| aerobic respiration | GO:0009060 | 11 (2.5%) | 0.03489 |
| cation transport | GO:0006812 | 28 (6.3%) | 0.03894 |
| nucleotide biosynthetic process | GO:0009165 | 14 (3.1%) | 0.03957 |
| purine-containing compound metabolic process | GO:0072521 | 15 (3.4%) | 0.04007 |

Enriched GO terms in differentially expressed genes were identified by Bonferroni Correction and FDR-corrected *P* < 0.05 as cut-off.
