# Supplementary material for: Phenotypes, antioxidant responses, and gene expression changes accompanying a sugar-only diet in Bactrocera dorsalis (Hendel) (Diptera: Tephritidae)
Source: BMC Evol Biol. 2017 Aug 17;17:194. doi: 10.1186/s12862-017-1045-5 (PMC5559826; doi:10.1186/s12862-017-1045-5)
Supplement: Supplementary file 7 — Selected GO terms significantly enriched for downregulated genes in SD versus ND. (DOCX 20 kb) [file 12862_2017_1045_MOESM7_ESM.docx]

**Additional file 7: Table S5** Selected GO terms significantly enriched for down-regulated genes in sugar-only diet (SD) versus normal diet (ND)

| **GO term** | **GO ID** | **DEGs (702)** | **FDR-corrected**  **(*P-value*)** |
| --- | --- | --- | --- |
| [chromosome organization](http://amigo.geneontology.org/amigo/term/GO:0051276) | GO:0051276 | 71 (10.1%) | 2.71e-20 |
| cell cycle | GO:0007049 | 119 (17.0%) | 6.48e-20 |
| nuclear division | GO:0000280 | 45 (6.4%) | 2.87e-12 |
| DNA metabolic process | GO:0006259 | 55 (7.8%) | 3.56e-12 |
| cell cycle process | GO:0022402 | 83 (11.8%) | 7.58e-12 |
| organelle fission | GO:0048285 | 45 (6.4%) | 7.63e-12 |
| mitotic nuclear division | GO:0007067 | 26 (3.7%) | 5.34e-11 |
| cellular macromolecule metabolic process | GO:0044260 | 265 (37.7%) | 1.00e-10 |
| DNA biosynthetic process | GO:0071897 | 15 (2.1%) | 1.30e-10 |
| nucleic acid metabolic process | GO:0090304 | 160 (22.8%) | 4.89e-10 |
| chromatin organization | GO:0006325 | 39 (5.6%) | 1.96e-09 |
| mitotic cell cycle | GO:0000278 | 63 (9.0%) | 5.68e-09 |
| single organism reproductive process | GO:0044702 | 98 (14%) | 1.75e-08 |
| DNA amplification | GO:0006277 | 11 (1.6%) | 3.62e-07 |
| negative regulation of macromolecule metabolic process | GO:0010605 | 41 (5.8%) | 6.22e-07 |
| organelle organization | GO:0006996 | 129 (18.4%) | 8.01e-07 |
| negative regulation of metabolic process | GO:0009892 | 44 (6.3%) | 9.10e-07 |
| negative regulation of gene expression | GO:0010629 | 34 (4.8%) | 1.51e-06 |
| nucleobase-containing compound metabolic process | GO:0006139 | 186 (26.5%) | 1.69e-06 |
| cellular process | GO:0009987 | 538 (76.6%) | 2.64e-06 |
| Reproduction | GO:0000003 | 112 (16.0%) | 4.99e-06 |
| reproductive process | GO:0022414 | 112 (16.0%) | 4.99e-06 |
| gamete generation | GO:0007276 | 80 (11.4%) | 8.87e-06 |
| organic cyclic compound metabolic process | GO:1901360 | 194 (27.6%) | 1.20e-05 |
| heterocycle metabolic process | GO:0046483 | 188 (26.8%) | 1.37e-05 |
| chromosome segregation | GO:0007059 | 23 (3.3%) | 1.52e-05 |
| cellular aromatic compound metabolic process | GO:0006725 | 189 (26.9%) | 2.72e-05 |
| gene silencing | GO:0016458 | 28 (4.0%) | 2.75e-05 |
| DNA replication | GO:0006260 | 20 (2.8%) | 5.41e-05 |
| regulation of metabolic process | GO:0019222 | 96 (13.7) | 6.35e-05 |
| female gamete generation | GO:0007292 | 64 (9.1%) | 6.93e-05 |
| cell cycle phase | GO:0022403 | 30 (4.3%) | 7.93e-05 |
| biological phase | GO:0044848 | 30 (4.3%) | 7.93e-05 |
| nuclear chromosome segregation | GO:0098813 | 18 (2.6%) | 8.12e-05 |
| cellular nitrogen compound metabolic process | GO:0034641 | 201 (28.6%) | 9.63e-05 |
| sister chromatid segregation | GO:0000819 | 16 (2.3%) | 0.00010 |
| chromatin modification | GO:0016568 | 23 (3.3%) | 0.00015 |
| cellular response to DNA damage stimulus | GO:0006974 | 31 (4.4%) | 0.00019 |
| covalent chromatin modification organization | GO:0016569 | 18 (2.6%) | 0.00025 |
| histone modification | GO:0016570 | 18 (2.6%) | 0.00025 |

Enriched GO terms in differentially expressed genes were identified by a Bonferroni Correction and FDR-corrected *P* < 0.05 as cut-off.
